# Heat Transfer and Entropy Generation Abilities of MWCNTs/GNPs Hybrid Nanofluids in Microtubes

**DOI:** 10.3390/e21050480

**Published:** 2019-05-09

**Authors:** Ahmed A. Hussien, Mohd Z. Abdullah, Nadiahnor Md Yusop, Wael Al-Kouz, Ebrahim Mahmoudi, Mohammad Mehrali

**Affiliations:** 1 Department of Mechanical Engineering, Faculty of Engineering, Al-Hussein Bin Talal University, P.O. Box 20, Ma’an, Jordan; 2 School of Mechanical Engineering, Engineering Campus, Universiti Sains Malaysia, Nibong Tebal 14300, Penang, Malaysia; 3 Faculty of Chemical Engineering, Jalan Ilmu 1/1, Universiti Teknologi MARA, Shah Alam 40450, Selangor, Malaysia; 4 Mechatronics Engineering Department, German Jordanian University, Amman 11180, Jordan; 5 Department of Chemical and Process Engineering, Universiti Kebangsaan Malaysia, Bangi 43600, Selangor Darul Ehsan, Malaysia; 6 Process and Energy Department, Delft University of Technology, Leeghwaterstraat 39, 2628 CB Delft, The Netherlands

**Keywords:** MWCNTs, GNPs, hybrid nanofluid, microtube, heat transfer coefficient

## Abstract

Massive improvements in the thermophysical properties of nanofluids over conventional fluids have led to the rapid evolution of using multiwalled carbon nanotubes (MWCNTs) and graphene nanoplatelets (GNPs) in the field of heat transfer. In this study, the heat transfer and entropy generation abilities of MWCNTs/GNPs hybrid nanofluids were explored. Experiments on forced convective flow through a brass microtube with 300 µm inner diameter and 0.27 m in length were performed under uniform heat flux. MWCNTs/GNPs hybrid nanofluids were developed by adding 0.035 wt.% GNPs to MWCNTs water-based nanofluids with mass fractions of 0.075–0.125 wt.%. The range of the Reynolds number in this experiment was maintained at Re = 200–500. Results showed that the conventional approach for predicting the heat transfer coefficient was applicable for microtubes. The heat transfer coefficient increased markedly with the use of MWCNTs and MWCNTs/GNPs nanofluids, with increased pressure dropping by 12.4%. Results further showed a reduction by 37.5% in the total entropy generation rate in microtubes for hybrid nanofluids. Overall, MWCNTs/GNPs hybrid nanofluids can be used as alternative fluids in cooling systems for thermal applications.

## 1. Introduction

Researchers have revealed that nanofluids, instead of conventional fluids, have been the new coolants for recent heat transfer applications. The development of heat transfer with nanofluids mainly depends on the enhancement of thermophysical properties because of suspended nanoparticles. However, hybrid nanofluids have shown further improvement in thermophysical properties and characteristics because of the synergy of two or more kinds of nanoparticle materials. This finding has motivated researchers to prepare novel hybrid nanofluids physically—by dispersing different prepared nanoparticle materials—or chemically—by synthesizing nanocomposite particles and then dispersing them in a base fluid [1,2,3,4,5]. 

Advanced technology in recent industrial applications emits high heat flux, which causes destruction that will lead to irreversibility and consequently lower efficiency. This phenomenon led to the use of microtubes because of their high heat fluxes with lower exergy destruction, and increased ratio of exposed surface area to heat per size [6,7,8,9]. 

Numerous experimental and numerical studies have focused on the heat transfer performance of microtubes [10,11], particularly on the applicability of conventional relations for predicting friction factor for laminar flow in microtubes. The role of nanofluids on the performance of heat transfer and pressure drop in microtubes has also been studied [12,13]. Different kinds of nanofluids with various microtube dimensions have been used to show heat transfer enhancement. Table 1 shows some of the recent investigations on convective heat transfer using nanofluids and microtubes and their findings. Nanofluids have minimized the entropy generation in thermal engineering systems and enhanced heat transfer. Based on the second law of thermodynamics, several studies have focused on the thermal performance of circular tubes with mono and hybrid nanofluids [14,15,16,17,18,19,20,21,22,23]. Table 2 summarizes the recent works conducted on the effects of nanofluids on entropy generation in thermal systems. Notably, mono and hybrid nanofluids have been used to reduce the irreversibility of different thermal applications and to minimize the lost work during the process. Many parameters affect the reduction rate of the total entropy generation rate, such as the construction of the thermal system, fluid velocity, as well as nanofluid concentrations and types.

As shown in the review above, studies on forced convection heat transfer using mono and hybrid nanofluids in microtubes are limited. In particular, the use of hybrid nanofluids in microtubes has never been tested before. Therefore, this work aimed to examine thermal performance and pressure drop using MWCNTs/water mono nanofluid and MWCNTs/GNPs water-based hybrid nanofluids flowing laminarly through the microtubes. Uniform heat flux was exposed to the outer surface with a constant inlet temperature. The experiment was performed with different weight concentrations of mono nanofluids (0.0%, 0.075%, and 0.125%). For the hybrid nanofluid, 0.035% of GNPs was added to the same previously used concentration.

## 2. Materials and Methods

### 2.1. Nanofluid Preparation and Properties

Commercial MWCNTs and GNPs were purchased from USAINS Infotech Sdn. Bhd. and XG Sciences, Inc. (Lansing, MI, USA), respectively. Table 3 shows the specifications of MWCNTs and GNPs.

To achieve stable MWCNTs nanofluids and MWCNTs/GNPs hybrid nanofluids, specific amounts of polyvinylpyrrolidone (PVP) were initially dissolved in distilled water using a magnetic stirrer at 60 °C. Different amounts of MWCNTs powder (0.075 and 0.125 wt.%) were then added and dispersed using a high-power probe sonicator (SG-24-500P, Telsonic Ultrasonics) for several minutes until the mixture was homogenous. To disperse 0.035 wt.% of GNPs in the MWCNT nanofluids, the previous procedure was repeated, but the sonication time was extended (see [9] for more details). This procedure was based on the research conducted by Fadhillahanafi et al. [31], whose findings revealed the importance of adding PVP to carbon nanotubes (CNTs). Moreover, Sadeghinezhad et al. [32] prepared stable GNP nanofluids via a high-powered ultrasonication probe.

**Figure 1:**
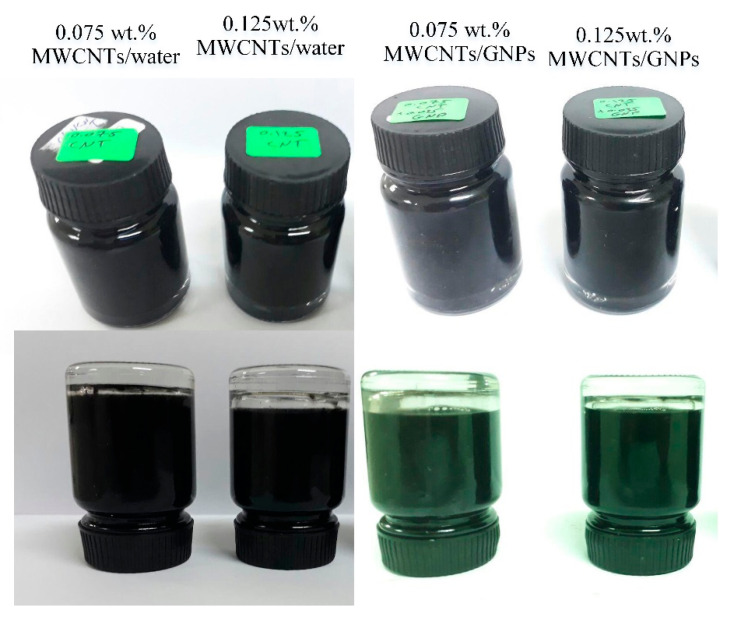
Photo after more than six months for the working nanofluids.

Furthermore, the stability of all samples which were used as working fluids in the experimental runs was tested by naked eyes. As shown in Figure 1, the mono nanofluids and hybrid nanofluids showed good stability even after six months.

**Figure 2:**
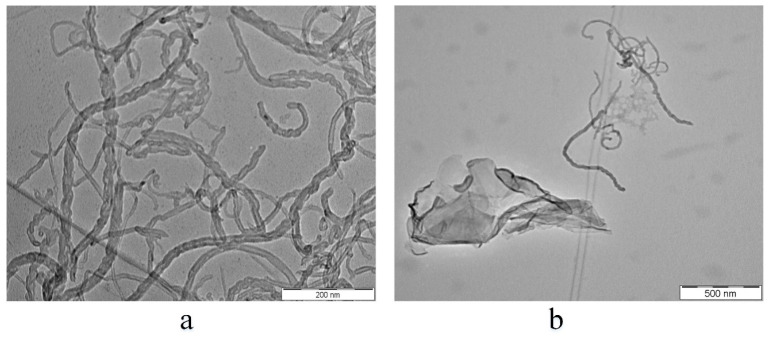
TEM images of (**a**) MWCNTs/water nanofluid and (**b**) MWCNTs/GNPs hybrid nanofluid.

Transmission electron microscopy (TEM) was used to characterize the mono and hybrid nanofluids shown in Figure 2a,b, respectively. The stability of nanofluids was observed by noting fewer agglomerations in these figures, even with large nanomaterials suspended in the base fluid. Moreover, the cylindrical shape of MWCNTs was noted, despite the long-term exposure to high-power sonication.

The most important thermophysical properties for convective heat transfer are density, heat capacity, viscosity, and thermal conductivity. The density and heat capacity of mono and hybrid nanofluids were predicted using a mixture model, as shown in the following Equations [2]:

(1) $$\rho_{hy}=\varphi_{np_{1}}\rho_{np_{1}}+\varphi_{np_{2}}\rho_{np_{2}}+\cdots +\varphi_{np_{n}}\rho_{np_{n}}+\left(1-\varphi_{np}\right)\rho_{bf}$$

(2) $$c_{hy}=\frac{\varphi_{np_{1}}\rho_{np_{1}}c_{np_{1}}+\varphi_{np_{2}}\rho_{np_{2}}c_{np_{2}}+\cdots +\varphi_{np_{n}}\rho_{np_{n}}c\rho_{np_{n}}+\left(1-\varphi_{np}\right)\rho_{bf}c_{bf}}{\rho_{hy}}$$

where $\varphi_{np}=\sum_{i=1}^{n}\varphi_{{np}_{i}}$, which is the total volume concentration. The volume concentration $\varphi_{v}$ can be converted to weight concentration $\varphi_{w}$ using Equation (3):

(3) $$\varphi_{v}=\frac{\varphi_{w}\rho_{bf}}{\rho_{p}+\varphi_{w}\rho_{bf}-\varphi_{w}\rho_{p}}.$$

**Figure 3:**
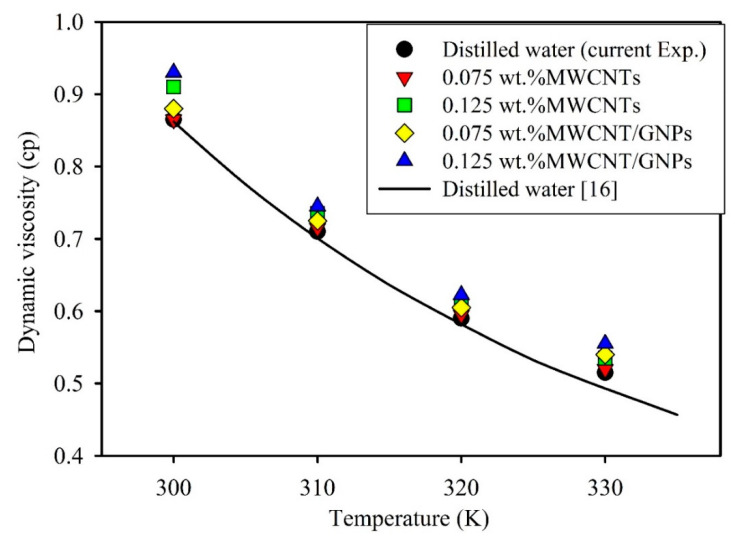
Viscosity versus temperature for MWCNTs nanofluids and MWCNTs/GNPs hybrid nanofluids.

The viscosities of mono and hybrid nanofluids were determined using a Brookfield viscometer (RVDV-III U, USA) at different temperatures, with accuracy reaching 2.7% on average, compared with one of the viscosity values of distilled water from a well-known source [33]. The fluid showed a similar behavior to that of any conventional fluid; for example, the viscosity decreased markedly with increased temperature. Figure 3 shows the viscosity at different concentrations of mono and hybrid nanofluids at varying temperature levels. The results indicate the increase in viscosity of existing nanomaterials on the base fluid rise, particularly with large nanomaterials. As shown in Figure 3, the hybrid nanofluids caused a significant increase in viscosity relative to that of distilled water upon reaching 6.4%. This result was due to the high flow resistance and friction force between fluid layers in addition to dissipation force, as well as the increased agglomeration probability of nanomaterials, particularly to the large MWCNTs and GNPs. High viscosity values of different nanofluids were observed compared with a model commonly used to predict the viscosity of nanofluids, that is, the Batchelor correlation [34], in Equation (4):

(4) $$\mu_{eff}\left(T\right)=\left(1+2.5\varphi_{v}+6.2\varphi_{v}{}^{2}\right)\mu_{bf}\left(T\right).$$

The thermal conductivity of nanofluids was predicted using the Hamilton and Crosser Model [35], which considers the shape effect of nanomaterials using the factor n, as shown in Equation (5):

(5) $$k_{eff}\left(T\right)=k_{bf}\left(T\right)\frac{k_{p}+\left(n-1\right)k_{bf}\left(T\right)+\left(n-1\right)\left(k_{p}-k_{bf}\left(T\right)\right)\varphi_{v}}{k_{p}+\left(n-1\right)k_{bf}\left(T\right)-\left(k_{p}-k_{bf}\left(T\right)\right)\varphi_{v}},$$

where n=6 for high aspect ratio nanoparticles [36].

However, for low concentrations of nanofluids, Duangthongsuk and Wongwises [37] revealed a negligible difference in heat transfer coefficient when using different conventional models in predicting the thermophysical properties of nanofluids.

### 2.2. Experimental Setup

**Figure 4:**
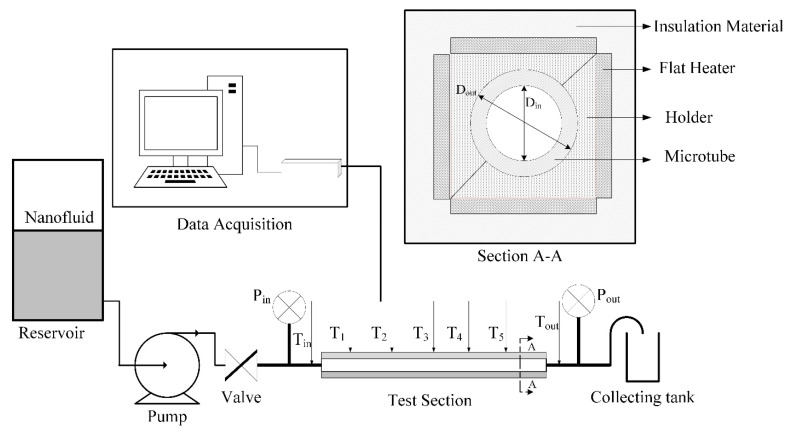
Schematic of the experimental setup and test section.

The experimental setup and test section in this study were proposed to achieve the objectives of testing the enhancement in heat transfer coefficient and pressure drop using microtubes via mono nanofluids and hybrid nanofluids. Figure 4 displays a schematic of the experimental setup and test section. The open loop flow setup was driven by a low flow-rate tubing pump (Cole-Parmer, USA), which was used to flow the nanofluids from the transparent tank through the test section prior to collection of the working fluid in the storage tank. The test section consists of a brass microtube, with inner and outer diameters of 300 and 500 µm, respectively. The microtube has a heated length of 27 cm and was placed inside a stainless-steel minitube holder. A heat sink compound (RS Components Ltd., USA) was utilized to fill the gap. Four flat heaters with a total power of 8.89 W were applied to the holder sides after five thermocouples (K-type) were attached onto the outer microtube wall at different axial locations (Z/D = 67, 200, 367, 533, and 833). A portable data acquisition module (Advantech Co., Ltd. Taiwan) was linked with all thermocouples to display the required temperature. Numerous layers of woven fiberglass were wrapped around the test section to reduce heat loss. Two tri-plastic pipe fittings were connected at the entrance and exit of the microtube, and a digital pressure indicator (DPI-705, Druck Ltd., UK) was installed to measure inlet and outlet pressure levels.

The inlet temperatures of all samples were fixed at 27 °C before taking the measurement values. Also, the range of average velocities of working fluids were varied between 0.39 to 1.26 m/s in order to get the desired Reynolds number. 

### 2.3. Data Reduction

The local-heat transfer coefficient h(Z) was predicted using the measurements of inlet and outlet temperatures and wall temperature at different axial locations (Z = 20, 60, 110, 160, and 250 mm) [33], as in Equation (6):

(6) $$h\left(Z\right)=\frac{q}{\left(T_{wi}\left(Z\right)-T_{f}\left(Z\right)\right)},$$

where q, $T_{f}\left(Z\right)$, and $T_{wi}\left(Z\right)$ are the actual heat flux gained by working fluids, mean fluid temperature, and inner wall temperature at specific distance Z, respectively, which can be calculated using the following equations:

(7) $$q=\rho_{nf}c_{p,nf}u\left(T_{in}-T_{out}\right),$$

(8) $$T_{wi}\left(Z\right)=T_{wo}\left(Z\right)+\frac{qln\left(\frac{D_{o}}{D_{i}}\right)}{2\pi lk_{s}},$$

(9) $$T_{f}\left(Z\right)=T_{in}+q\pi D_{i}Z/\left(\rho_{nf}c_{p,nf}uA\right),$$

where $T_{wo}\left(Z\right),$ $k_{s}$, and l are the surface wall temperature, thermal conductivity of the brass material, and length of minitube, respectively. In addition, u is the bulk velocity, which can be computed from the flow rate Q and microtube cross-section area A as u=Q/A. 

The Nusselt number (Nu) was estimated using Equation (10):

(10) $$Nu\left(Z\right)=h\left(Z\right)D_{i}/k_{nf}.$$

The percentage of the enhancement heat transfer coefficient was calculated using Equation (11):

(11) $$h_{enhanc.}\%=\frac{\left(h_{nf}\left(z\right)-h_{water}\left(z\right)\right)}{h_{water}\left(z\right)}\times 100\%.$$

The thermal entropy generation rate (${\dot{S}}_{th}$) was defined as:

(12) $${\dot{S}}_{th}=\frac{\pi D_{i}^{2}lq^{2}}{k_{nf}NuT_{av}}.$$

This can also be introduced in terms of heat transfer coefficients as follows:

(13) $${\dot{S}}_{th}=\frac{\pi D_{i}lq^{2}}{hT_{av}},$$

where $T_{av}=\frac{T_{in}-T_{out}}{ln\left(\frac{T_{in}}{T_{out}}\right)}$.

The friction entropy generation rate (${\dot{S}}_{fr}$) can be determined as follows [23]:

(14) $${\dot{S}}_{fr}=\frac{32{\dot{m}}^{3}fl}{\pi^{2}\rho^{2}T_{av}D_{i}^{5}}.$$

The uncertainty analysis for all measurements was performed by the Taylor method [38], which is based on the deviations of repeated measurements from the mean and the number of iterated values. The maximum uncertainties of all the derived experimental parameters are <5.5%. In addition, the average data of three runs were used to perform all experiments for distilled water, MWCNTs, and MWCNTs/GNPs hybrid nanofluids. Table 4 shows the precision values of the used apparatus in the present experiments.

## 3. Results and Discussion

### 3.1. Validation and Verification

**Figure 5:**
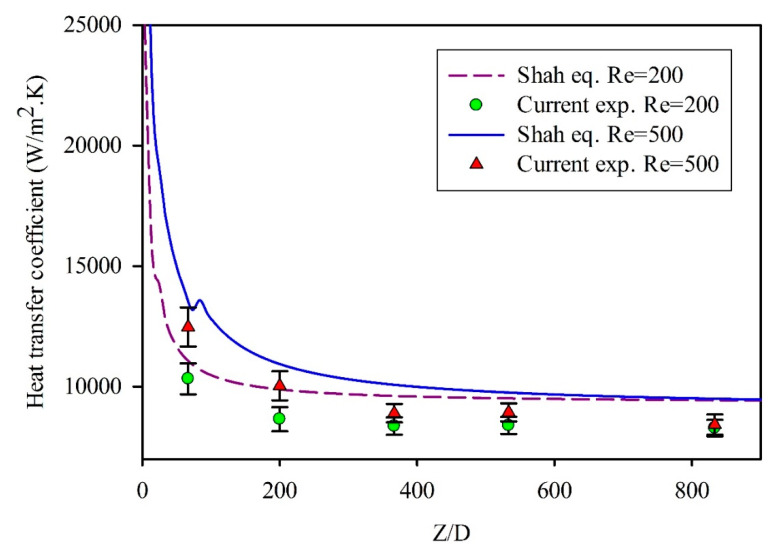
Comparison of the local heat transfer coefficients of distilled water along the tube with the Shah equation for different Reynolds numbers.

Distilled water was used to validate the experimental results. The heat transfer coefficients and pressure drop values at different Reynolds numbers were compared with the well-known Equation (15). Figure 5 shows the comparison between the values of local heat transfer coefficient at different Reynolds numbers and Shah equation [39]:

(15) $$Nu=\{\begin{array}{ll} 1.953Z^{{*}^{\frac{1}{3}}} & Z^{*}>33.33\\ 4.364+0.0722Z^{*} & Z^{*}\leq 33.33 \end{array},$$

where $Z^{*}=RePr\left(\frac{D_{in}}{Z}\right)$ and $Pr=\frac{c_{p,nf}\mu_{nf}}{k_{nf}}$.

The experiment rig results show average deviations of 9.4% and 8% at Re = 200 and 500, respectively. These experimental errors are highly accepted especially when using small tubes [12,40]. Notably, the values of the local heat transfer coefficient are slightly less than theoretical values. This difference is due to conjugated heat transfer, which is caused by the reduction in the local Nusselt number at the entrance and exit regions [41]. This phenomenon has also been demonstrated by Salman et al. [13] at a low Reynolds number. 

The pressure drop (Δp) of a fully developed laminar flow and smooth wall tube can be estimated by applying Darcy’s law:

(16) $$\Delta p=\frac{f\rho lu^{2}}{2D_{in}},$$

where f is friction factor, which can be calculated considering the Hagen–Poiseuille formula:

(17) $$f=\frac{64}{Re}.$$

**Figure 6:**
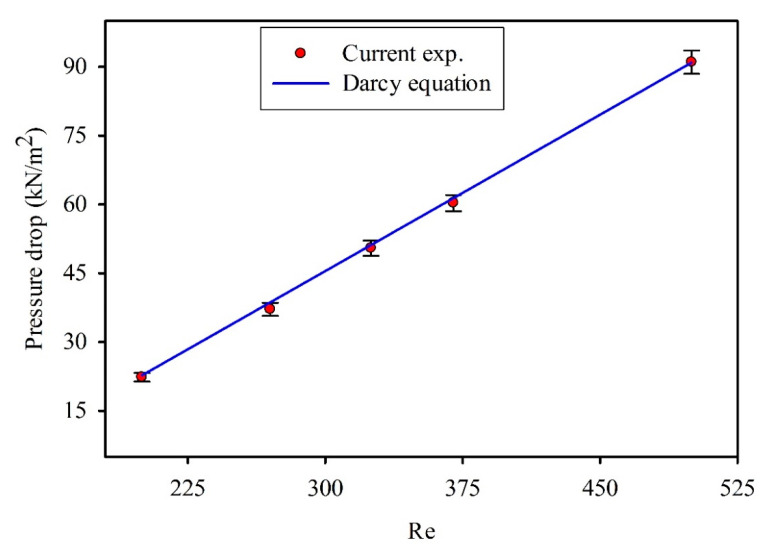
Comparison of pressure drop at different Reynolds numbers using the Darcy equation.

Figure 6 shows consistent results of pressure drops with distilled water at various Reynolds numbers between testing values and the Darcy equation. The deviation between experiment results and conventional correlation for fully laminar flow was minor, showing an average value of <2%, which was due to contraction and expansion loss [42]. This result indicates that the conventional correlations can predict the pressure drop in the microtubes [10,11].

### 3.2. Heat Transfer Characteristics and Pressure Drop

**Figure 7:**
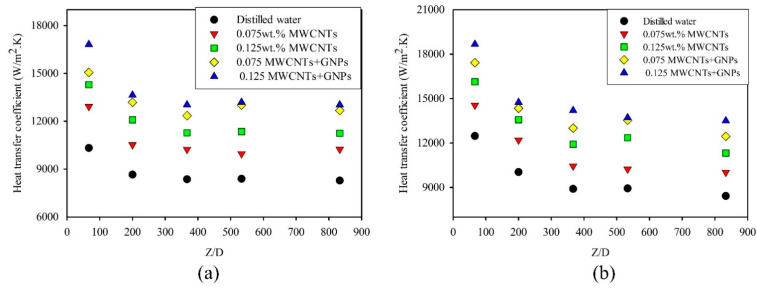
Local heat transfer coefficient along microtubes at varying weight concentrations of mono nanofluids and hybrid nanofluids, at (**a**) Re = 200 and (**b**) 500.

**Figure 8:**
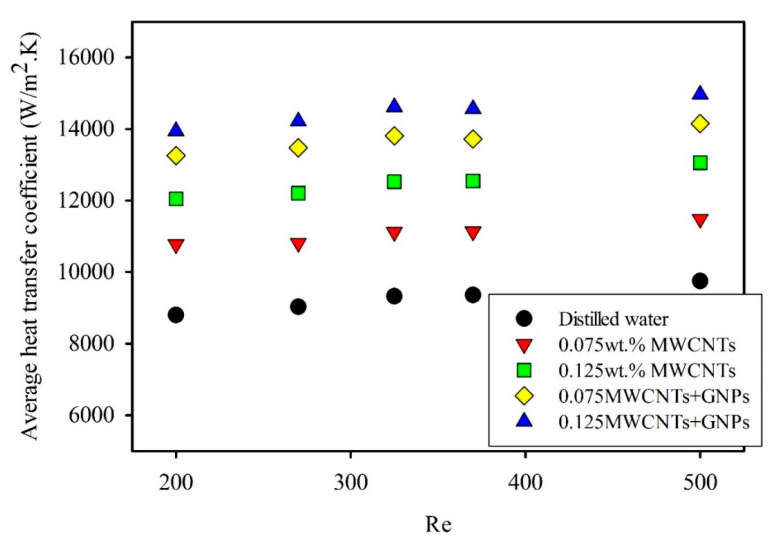
Average heat transfer coefficients at different Reynolds numbers for different weight concentrations of mono nanofluids and hybrid nanofluids.

In conventional theory, the local Nusselt numbers in laminar flow and under uniform heating are high at the entrance of the tube and eventually decrease until a specific value, 4.36, is reached. Thus, a similar effect on the heat transfer coefficient was observed. The maximum values were due to the high transfer rate at the developing region. Figure 7 displays the variation in heat transfer coefficient along the axial microtube length at different Reynolds numbers for mono nanofluids and hybrid nanofluids. These figures indicate an increased heat transfer coefficient for all samples with an increased Reynolds number, as well as decreased value with axial direction. Evidently, the heat transfer coefficient improved with increased weight concentration for both mono and hybrid nanofluids. The enhancement of heat transfer coefficient increased with the Reynolds number. The mean velocity of the working fluids increased, which directly affects the elevation in heat transfer rate between working fluids and microtube wall. For example, the local heat transfer coefficient at Z/D = 200 was 8650 W/m^2^·K at Re = 200. At Re = 500, the local heat transfer coefficient was 10,030 W/m^2^·K (Figure 8).

The heat transfer coefficients relative to all nanofluid samples were at the peak value compared with distilled water. The presence of high thermal conductivity nanomaterials in nanofluids was the primary cause for the increased heat transfer coefficients. Moreover, literature shows that nanoparticle migration, such as Brownian motion, inside the microtube causes more interaction between nanoparticles and the tube wall. In addition to non-uniformity in nanoparticle concentrations, this motion led to an increased Nusselt number and heat transfer coefficient [43]. Buongiorno [44] explained that abnormal thermal conductivity increases with nanofluids, which develops the heat transfer coefficient by a significant variation of nanofluid properties within the boundary layer because of temperature gradient and thermophoresis.

**Figure 9:**
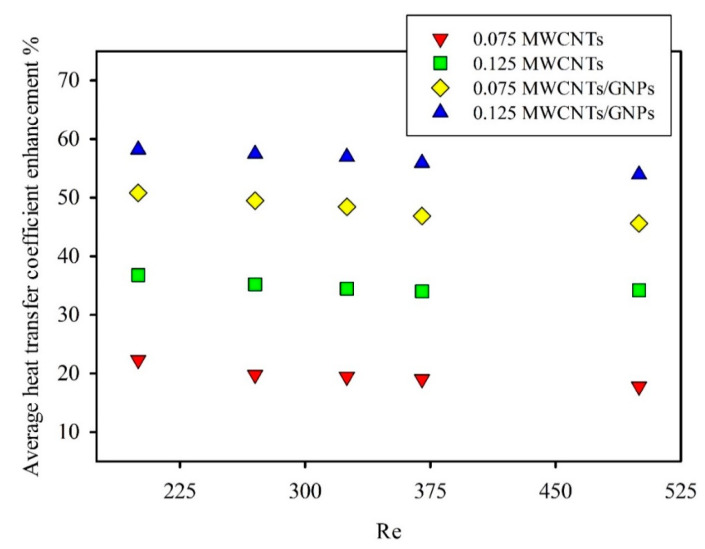
Average enhancement of the heat transfer coefficient compared with that of distilled water at different Reynolds numbers for different weight concentrations of mono nanofluids and hybrid nanofluids.

The presence of GNPs at a low concentration of 0.035 wt.% significantly enhanced the heat transfer coefficient. As shown in Figure 7, the values of the heat transfer coefficient for hybrid nanofluids clearly exceeded those of mono nanofluids and distilled water. This high heat transfer coefficient was due to the role of GNPs in developing the thermophysical properties by high thermal conductivity; moreover, the unique properties of GNPs dispersed in base fluids can improve the forced convection heat transfer [45,46]. In addition, the actual heat transfer area between GNPs and distilled water increased because of their two-dimensional shape. All of these parameters considerably increased the heat transfer coefficient, as shown in Figure 9. The result showed a maximum enhancement of 58.2% with the 0.125wt.% MWCNT/GNP hybrid nanofluid, compared to 36.8% with the same weight concentration of mono nanofluids at Re = 200. The effect of adding low weight concentration of GNPs on average enhancement of heat transfer coefficient can be shown in Figure 9. For example, the average enhancement in heat transfer coefficient of 0.075 wt.% MWCNTs/GNPs (1.1 wt.% of nanoparticles) is found to be 48.4% at Re = 325, while for 0.125 wt.%MWCNTs nanofluid, the enhancement was 34.4%. Notably, the results did not show the role of different Reynolds numbers in any abnormally enhanced heat transfer coefficient. On the contrary, the figure shows a slight lack in average enhancement of heat transfer coefficient with an increased Reynolds number for all nanofluids. For instance, the average enhancement of the heat transfer coefficient for 0.125 wt.% MWCNT nanofluids was reduced from 36.8% at Re = 200 to 34.2% at Re = 500. The literature also mentioned the negative effect of the Reynolds number on the average enhancement in heat transfer via nanofluids. Ding et al. [47] observed a reduction in the average enhancement with 0.1 wt.% CNT/water nanofluids from 62% at Re = 800 to 58% at Re = 1000. Grag et al. [48] conducted an experiment on the effect of 0.1% MWCNT/water nanofluid on heat transfer performance, and the average enhancement decreased from 26% at Re = 600 to 18% at Re = 1200.

**Figure 10:**
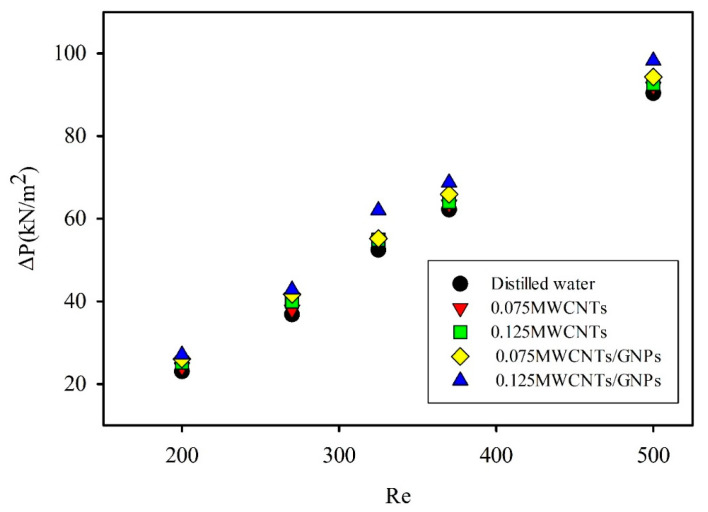
Pressure drop at different Reynolds numbers for varying weight concentrations of mono nanofluids and hybrid nanofluids.

Figure 10 illustrates the differences between inlet and outlet pressures at different Reynolds numbers for varying weight concentrations of mono and hybrid nanofluids. Notably, the values of pressure drop increased with an increased Reynolds number or nanofluid mean velocity, which caused high wall shear stresses. This connection was the same as conventional relations in macrotube flow. Moreover, the pressure drop of mono and hybrid nanofluids was higher than that of distilled water. This result is due to the presence of nanomaterials, which led to increased density and viscosity compared with distilled water for particular GNPs. The experimental records revealed that the addition of GNPs to MWCNT/water nanofluids increased the pressure drop even at low concentrations. For example, for 0.125 MWCNT, the average increase in pressure drop relative to that of the base fluid was 5.2%, and reached 12.4% with the addition of GNPs. This finding indicates a limitation in the use of GNPs in microtubes, that is, extra pumping power is required for flowing hybrid nanofluids. The following formula can be used to compute the pumping power [49]:

(18) $$P_{P}=\frac{\Delta P\dot{m}}{\rho_{nf}}.$$

**Figure 11:**
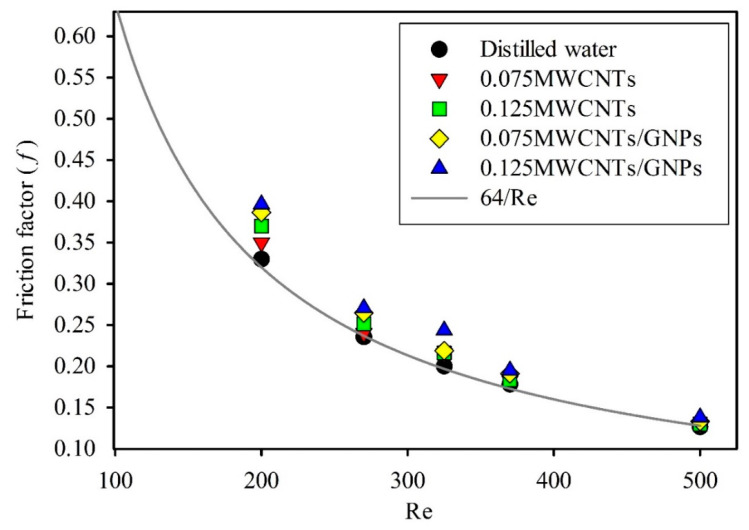
Friction factor at different Reynolds numbers for varying weight concentrations of mono and hybrid nanofluids.

The friction factors obtained from the pressure drop values using Equation (16) were contrasted with the theoretical laminar flow friction factor, as shown in Figure 11. The friction factor for all working fluids exceeded 64/Re but showed the same trend. The high values of friction factor accompanying the use of carbon nanomaterial-based hybrid nanofluids were the main obstacles for their application in the industrial sectors. Sundar et al. [50] noted the 1.18 penalty on pumping power for 0.3% volume concentrations of MWCNT–Fe_3_O_4_ water-based hybrid nanofluid. The increase in friction factor for flowing hybrid nanofluids in tubes is mainly due to the increase in viscosity [2]. In this experiment, a 6.2% increase in viscosity may be due to the 12.4% rise in friction factor compared with that of distilled water. However, the increase in friction factor in comparison with the enhancement of heat transfer coefficient was irrelevant.

### 3.3. Entropy Generation Rate Analysis

**Figure 12:**
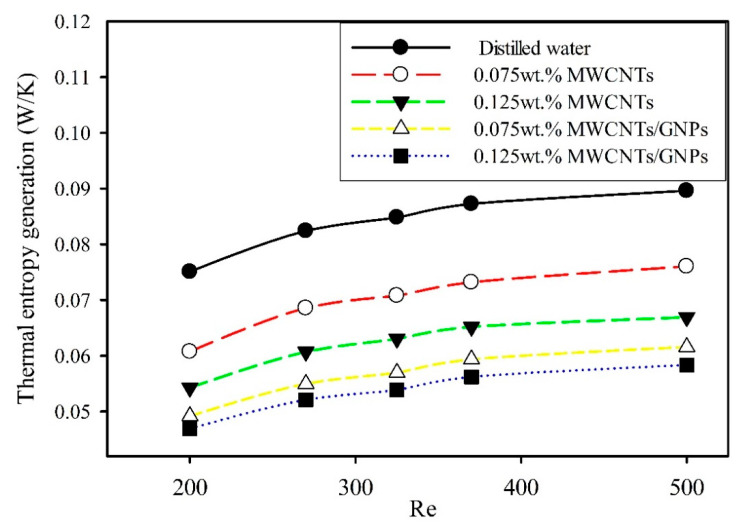
Thermal entropy generation rate of mono and hybrid nanofluid as a function of the Reynolds number.

In the current experimental study, the second law of thermodynamics was used to evaluate the thermal and frictional entropy generation rate performance of using MWCNTs/GNPs in microtubes. Figure 12 presents the thermal entropy generation in microtube with Reynolds numbers. The result shows the improvement of mono and hybrid nanofluids over the base fluid by reducing thermal entropy generation. As the weight concentrations of mono and hybrid nanofluid increase, thermal entropy generation decreases. For example, thermal entropy generation in microtubes for 0.125 wt.% MWCNTs and 0.125 wt.% MWCNTs + 0.035wt.% of GNPs at Re = 500 are 25% and 35% less than distilled water, respectively. However, the maximum reduction of thermal entropy generation in microtubes was 37.5% for 0.125wt.% MWCNTs/GNPs hybrid nanofluids at Re = 200.

Studies on thermal-generation rate in horizontal tubes confirm that thermal entropy generation decreases with nanofluids. The increase in bulk temperature and the heat transfer coefficient in microtubes, which is observed when using nanofluids, leads to reduced thermal entropy generation [14,21]. Figure 12 also shows increased thermal entropy generation with an increased Reynold number. This phenomenon is due to enhanced heat transfer coefficients in microtubes for low Reynolds numbers. Mehrali et al. [23] proved that thermal entropy generation increases as Reynolds numbers increase at low flow velocities Re ≤ 600, while it decreases at high Re. Li and Kleinstreuer [14] showed that operational Reynolds number values are related to the typical microstructure heating system. Therefore, using MWCNTs/GNPs in microtubes reduced the level of total irreversibilities, which can enhance the performance of hybrid nanofluids in cooling systems.

**Figure 13:**
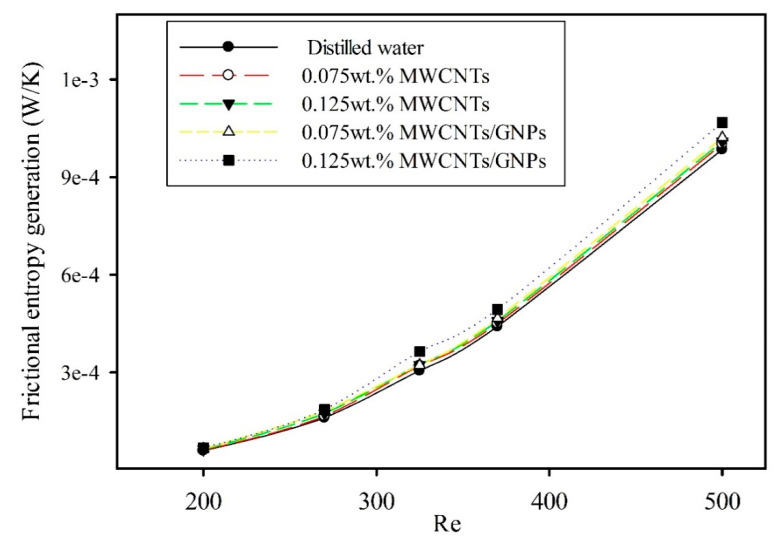
Frictional entropy generation rate of mono and hybrid nanofluid as a function of the Reynolds number.

Figure 13 presents the frictional entropy generation rate of mono and hybrid nanofluids as a function of Reynolds numbers. The resulting values showed increased frictional entropy generation with the Reynolds number and the existence of nanomaterials in the base fluid. This result was due to an increase in both the mass flow rate and viscosity of the fluids. Notably, the effect of frictional entropy generation on the total entropy generation in microtubes for mono and hybrid nanofluids was negligible. The reason for this is the low mass flow rate for the working fluids, due to the small tube diameters. The frictional entropy generation became significant with an increased fluid inlet Reynolds number [14]. Thus, overall, the main contribution of total entropy generation was due to thermal effect rather than the viscous effect on entropy generation.

## 4. Conclusions

Forced convective heat transfer, pressure drop, and entropy generation rate were explored experimentally using laminar flows of MWCNTs mono nanofluid and MWCNTs/GNPs hybrid nanofluid in microtubes with inner diameters of 300 µm. Customized weight concentrations were utilized in the experiment trials. In the experiment with distilled water, conventional correlations for predicting the heat transfer coefficient and pressure drop were applicable in microtubes with D_i_ = 300 µm. Results indicated a considerable increment in heat transfer coefficient for nanofluids compared with the base fluid, which was directly proportional to the nanoparticle concentration. The addition of a low concentration of GNPs to MWCNTs nanofluids caused obvious increments in the heat transfer coefficient. Results show that enhancing heat transfer is inversely related to the Reynolds number due to the decrease in thermal conductivity enhancement of the lower bulk temperature of the fluid, which decreases with an increase in Reynolds number. The highest average enhancement in heat transfer coefficients and reduction of total entropy generation were noted in 0.125 wt.% MWCNTs/GNPs hybrid nanofluid at approximately 58% and 37.5% compared with that of distilled water at Re = 200, respectively. Interestingly, increased pressure drop was observed when nanofluids were used, particularly with hybrid nanofluids, because of higher values of viscosities compared with those of mono nanofluids and the base fluid. Finally, the development of the heat transfer rate with the increased pressure drop was found to be insignificant.

## Figures and Tables

**Table 1:** Recent investigations of convective heat transfer using microtube.

Reference	Type of Study	Working Fluids	Microtube Di	The Key Finding
Li et al. [24]	Experimentally and numerically	Distilled water	D_i_ = 50–1570 µm	The conventional correlation is valid for predicting friction factor in smoothed microtubes
Sara et al. [10]	Experimentally	Distilled water	D_i_ = 200 µm	Poiseuille flow theory for predicting friction factor for laminar flow in microtubes remains valid.
Salaman et al. [25]	Numerically	Al_2_O_3_, CuO, SiO_2_ and ZnO/water	D_i_ = 50 µm	The Nusselt number for all cases increases with the volume fraction
Kurtoglu [12]	Experimentally	Fe_3_O_4_/water	D_i_ = 514 µm	The use of ferrofluid caused a 100% decrease in maximum surface temperature compared with the base fluid.
Yang and Lin [11]	Experimentally	Distilled water	D_i_ = 123 µm	Conventional relations are applicable for sizes exceeding 123 µm
Salman et al. [13]	Experimentally and numerically	Al_2_O_3_ and SiO_2_/water	D_i_ = 1 mm	The maximum heat transfer enhancement of 22% was pointed, and this percentage matched conventional correlations.
Hussien et al. [26]	Numerically	Al_2_O_3_ +graphene/water	D_i_ = 800 µm	The maximum heat transfer enhancement was for hybrid nanofluid with extra penalty of pressure drop
Ramirez-Tijerina et al. [27]	Numerically	Al_2_O_3_, TiO_2_, CuO, SiO_2_ and ZnO/water	D_i_ = 500 µm	Nusselt number increases with increase in Reynolds number and particle concentration
Khoshvaght-Aliabadi et al. [28]	Experimentally	Cu/water	D_i_ = 787 µm	Results of straight horizontal microtube are valid with conventional correlations.

**Table 2:** Effect of nanofluids on entropy generation in the thermal systems.

Reference	Type of Study	Working Fluids	Thermal Application	The Key Finding
Li and Kleinstreuer [14]	Numerically	CuO/water	Microchannel heat sink	Nanofluids can reduce entropy generation due to high thermal conductivity.
Singh et al. [15]	Analytically	Al_2_O_3_/water	Microchannel, Minichannel	The entropy generation rate depends on tube diameter for both laminar and turbulent flow.
Sohel et al. [17]	Analytically	Cu, Al_2_O_3_/water, EG	Microchannel, Minichannel	Smaller diameter produced less entropy generation. Nanofluids caused reduction of thermal entropy.
Mahian et al. [18]	Experimentally	Al_2_O_3_/water	Solar Collector	The entropy generation decreases with increasing the nanofluid concentration.
Ghanbarpour and Khodabandeh [19]	Experimentally	TiO_2_,Al_2_O_3_/water	Cylindrical heat pipe	Nanofluids reduced the entropy generation and improved thermal performance.
Mehrali et al. [20]	Experimentally	GNP/water	Circular tube	The total entropy generation decreases with increasing nanoparticle concentration.
Ahammed et al. [21]	Experimentally	Graphene+Al_2_O_3_/water	Minichannel heat exchanger	Pure graphene–water nanofluid shows higher thermal performance than hybrid nanofluids.
Mehrali et al. [23]	Experimentally	graphene/Fe_3_O_4_	Tube subjected to magnetic fields	Total entropy generation decreased when using hybrid ferro fluids.
Karimzadehkhouei et al. [29]	Experimentally	MWCNTs/water	horizontal minitubes	A reduction in the entropy generation rate compared to pure water was observed at Re = 500 for mass fractions of 0.25 and 0.5 wt.%
Ji et al. [30]	Numerically	Al_2_O_3_/water	Circular tube	The entropy generations decrease before the intersection points of nanofluids

**Table 3:** MWCNTs and GNPS specifications.

MWCNTs			GNPs		
Property	Unit	Value	Property	Unit	Specification
Average outer diameter	nm	15 ± 2	Typical surface area	m^2^/g	120 to 150
Average length	µm	1–5	Particles thickness and diameter	nm, µm	6–8 and 5–25
Carbon purity	%	>95	Carbon purity	%	>99.5

**Table 4:** Uncertainties of the experimental apparatus.

Apparatus	Uncertainty
K Type thermocouples	±0.2 °C
Balance	±0.0001
AC source	±3 Volts
Timer	±0.01 s
Collecting Beaker	±2 mL
Pressure Transducer	±7 pa
Brookfield Viscometer	±2%

## Nomenclature

A	Tube cross section area (m^2^)
$c_{p}$	Specific heat (J/kg·K)
D_in_	Tube inner diameter (µm)
$D_{o}$	Tube outer diameter (µm)
*f*	Darcy friction factor
*h*	Heat transfer coefficient (W/m^2^·K)
*k*	Thermal conductivity (W/m·K)
*l*	Tube length (cm)
*Nu*	Nusselt number
Δ*p*	Pressure drop (N/m^2^)
*Pr*	Prandtl number
*Q*	Volume flow rate (m^3^/s)
*q*	Heat flux (W/m^2^)
*Re*	Reynolds number
$\dot{S}$	Entropy generation rate (W/m·K)
$T_{wi}$	Inner wall temperature (K)
$T_{wo}$	Outer wall temperature (K)
*u*	Bulk fluid velocity (m/s)
**Greek Symbols**	
ρ	Density (kg m^−3^)
µ	Dynamic viscosity (N s m^−2^)
*φ*	Nanoparticles fraction (%)
**Subscripts**	
*av*	Average
*bf*	Base fluid
*eff*	Effective
*fr*	Friction
*hy*	Hybrid nanofluids
*nf*	Nanofluid
*np*	Nanoparticles
*th*	Thermal
